# Monitoring of efficacy and safety of artemisinin-based anti-malarials for treatment of uncomplicated malaria: a review of evidence of implementation of anti-malarial therapeutic efficacy trials in Tanzania

**DOI:** 10.1186/s12936-015-0649-8

**Published:** 2015-03-29

**Authors:** Alex Shayo, Joram Buza, Deus S Ishengoma

**Affiliations:** The Nelson Mandela African Institution of Science and Technology, P.O Box 447, Arusha, Tanzania; National Institute for Medical Research, Tanga Research Centre, P.O Box 5004, Tanga, Tanzania

**Keywords:** Combination therapy, Artemether, Lumefantrine, Artesunate, Amodiaquine, Efficacy, Safety, *Plasmodium falciparum* and Tanzania

## Abstract

**Background:**

Prompt diagnosis and effective treatment are considered the cornerstones of malaria control and artemisinin-based combination therapy (ACT) is currently the main anti-malarial drugs used for case management. After deployment of ACT due to widespread parasite resistance to the cheap and widely used anti-malarial drugs, chloroquine and sulphadoxine/pyrimethamine, the World Health Organization recommends regular surveillance to monitor the efficacy of the new drugs. The present paper assessed the implementation of anti-malarial efficacy testing for monitoring the therapeutic efficacy of ACT for treatment of uncomplicated malaria in Tanzania before and after policy changes in 2006.

**Methods:**

A literature search was performed for published clinical trials conducted in Tanzania from 2001 to 2014. It focused on studies which assessed at least one form of ACT for treatment of uncomplicated falciparum malaria in children less than 10 years and reported efficacy and safety of the tested anti-malarials. References were imported into the Endnote library and duplicates removed. An electronic matrix was developed in Microsoft Excel followed by full text review with predetermined criteria. Studies were independently assessed and information related to ACT efficacy and safety extracted.

**Results:**

Nine papers were selected from 125 papers screened. The efficacy of both artemether-lumefantrine (AL) and artesunate-amodiaquine (AS + AQ) against uncomplicated *P. falciparum* infections in Tanzania was high with PCR-corrected cure rates on day 28 of 91-100% and 88-93.8%, respectively. The highest day-3 parasite positivity rate was 1.4%. Adverse events ranged from mild to serious but were not directly attributed to the drugs.

**Conclusion:**

ACT is efficacious and safe for treatment of uncomplicated malaria in Tanzania. However, few trials were conducted in Tanzania before and after policy changes in 2006 and thus more surveillance should be urgently undertaken to detect future changes in parasite sensitivity to ACT.

## Background

Malaria is by far the most important parasitic disease in Tanzania and in other tropical countries, causing loss of life and morbidity with more than three billion people at risk globally [1]. Prompt diagnosis and effective treatment are considered the cornerstones of malaria control [2-4]. However, resistance of malaria parasites to the cheap and commonly used anti-malarials has become a major challenge to malaria control. Worldwide resistance of *Plasmodium falciparum* to chloroquine (CQ) and the rapid spread of resistance to sulphadoxine-pyrimethamine (SP) prompted the introduction of artemisinin combination therapy (ACT). In 2001, a World Health Organization (WHO) expert panel recommended use of ACT for treatment of uncomplicated falciparum malaria in all endemic countries [5].

The combination therapy involves simultaneous use of two or more blood schizonticidal drugs with independent modes of action and different biochemical targets in the parasite, a mechanism which delays development of parasite resistance [6,7]. ACT can be either fixed-combination medicinal products, in which different drugs are co-formulated in the same tablets or capsules, or multiple drug therapy, in which the components are co-administered in separate tablets or capsules. The short-lived artemisinin-derivative component of ACT causes rapid and effective reduction of parasite biomass and gametocyte carriage, while the partner drug with a longer duration of action clears the remaining parasite biomass. The two drugs work together to achieve effective clinical and parasitological cure and protect each other from development of resistance by *P. falciparum* [5].

The current recommended combinations are artemether-lumefantrine (AL), artesunate-amodiaquine (AS + AQ), artesunate-mefloquine (AS + MQ), dihydroartemisinin-piperaquine (DHA + PQ), and artesunate-sulphadoxine/pyrimethamine (AS + SP) [8]. Artemisinin-naphthoquine combination (ARCO™) has also been tested and has shown some potentials as a new generation ACT for the treatment of uncomplicated malaria, but it is still under further clinical evaluation [9]. Prior to policy changes, malaria-endemic countries had to choose among the above combinations based on different factors, such as price, level of parasite resistance to the partner drug in the local parasite population and the capacity of the country to sustainably supply the anti-malarials [8,10-12]. Thus, AL and AS + AQ are the main ACT that have been widely deployed in majority of the African countries [13].

Parasite resistance to anti-malarials is of great concern in the efforts to control malaria worldwide. The parasites develop resistance by initially becoming tolerant to the drugs before they become fully resistant. The resistant parasites have an ability to survive under therapeutic levels of anti-malarial drugs which would otherwise kill both sensitive and tolerant parasites [14]. Parasite resistance to the anti-malarials can be assessed through in vivo, in vitro tests, analysis of known molecular markers of parasite resistance and by measurement of drug levels among patients treated with the respective anti-malarials [15-18]. Measures such as parasite clearance time, fever clearance time or gametocyte clearance time in in vivo and in vitro assays are used to indirectly detect any variation in parasite sensitivity thereby facilitating early warning in case of emergence of tolerance or resistance [19-21].

In vivo response of patients to treatments provides more information to clinicians and policy makers and is considered the gold standard for assessing anti-malarial efficacy. However, therapeutic efficacy must be interpreted as an interaction between the host factors (e.g., age, immunity and pharmacogenetics), the parasite factors (e.g., biomass, resistance) and the drug factors (e.g., pharmacokinetic properties, drug quality etc.) [17]. In vitro and molecular studies on the other hand, are useful in providing addition information on the parasite susceptibility without confounding effects of host factors although it is difficult to judge their clinical relevance [17]. It is only recently that K13-propeller polymorphism has been documented as a molecular marker for monitoring artemisinin resistance [22] and simpler genotyping protocols will be required before it can be fully adopted as a routine surveillance tool in most endemic countries with limited technical and infrastructural resources. Thus, effective monitoring of ACT has been and will continue to rely largely on in vivo studies with adequate follow-up. However, in vivo efficacy studies have logistics and cost implications which have limited their regular implementation.

WHO recommends regular efficacy testing for monitoring the efficacy of anti-malarials [2,8,23]. In Tanzania, the national malaria control programme (NMCP), in collaboration with its partners, including research institutions, medical universities, WHO country office and others, including funding agencies, have been conducting regular therapeutic efficacy trials (TETs). The efforts of the NMCP to ensure regular TETs have also been complemented by trials conducted by independent researchers. Thus, regular implementation of TETs is one of the priority activities of the Tanzania NMCP, which provides useful data for monitoring the efficacy of ACT and detecting emergence of drug tolerance/resistance to these and other anti-malarials used in the country. The findings of these studies have been used to guide the NMCP in reviewing and changing anti-malarial drug policy in the past [24,25].

Tanzania changed its malaria treatment policy from CQ to SP monotherapy as the first-line drug for the treatment of uncomplicated malaria in 2001 [25]. However, shortly after its introduction, *P. falciparum* resistance to SP was reported [26,27] and this forced the country to change the guidelines in 2006 [24] to introduce ACT. Studies conducted in 2004 in Tanzania indicated that the mean SP treatment failure was as high as 25.5% [28] which was higher than the WHO recommended cut-off failure rate (15%), above which policy changes have to be made [2,29]. Whereas Zanzibar adopted AS + AQ as first-line treatment in November 2001 [30], Tanzania mainland adopted AL as first-line anti-malarial treatment of uncomplicated falciparum malaria in November 2006 and became fully rolled out in January 2007 [24].

Unfortunately, artemisinin-resistant field isolates have been reported recently in four countries of Southeast Asia (Cambodia, Myanmar, Thailand, and Vietnam) and threatens the current progress in controlling the disease [1,2,19]. There is a potential for such isolates to spread to other malaria-endemic regions, including sub-Saharan countries (SSA) as happened with previous anti-malarials [31,32], and such parasites might be extremely difficult to control. Such threat underscores the importance of intensive surveillance of artemisinin resistance to prevent the spread of resistance to other countries, as recommended within the WHO Global Plan for artemisinin resistance containment (GPARC) [23]. Such surveillance facilitates early detection of emergence and spread of tolerance/resistance to ACT and provides evidence for formulating mitigation and containment strategies as recommended by WHO [23,33], thus helping to safeguard the long-term usefulness of these drugs.

The present paper reviewed the implementation of in vivo efficacy testing in Tanzania before and after deployment of ACT in order to monitor the efficacy of ACT for the treatment of uncomplicated malaria. The paper compares the cure rates, parasite clearance and fever clearance times and safety data reported in clinical trials involving ACT in Tanzania that were published between 2001 and 2014. It provides updates on country-specific performance of ACT after its wide-scale deployment for treating uncomplicated falciparum malaria.

## Methods

Published literature was searched and it involved papers published from January 2001 to August 2014. English language articles indexed in PubMed were searched using search terms: ‘Tanzania AND malaria AND artemether-lumefantrine’, ‘Tanzania AND malaria AND artesunate-amodiaquine’, ‘Tanzania AND malaria AND artesunate-mefloquine’, ‘Tanzania AND malaria AND dihydroartemisinin-piperaquine’ and ‘Tanzania AND malaria AND artesunate-sulphadoxine-pyrimethamine’. PubMed was used for primary search but in addition, Google Scholar, the Worldwide Antimalarial Resistance Network (WWARN) standardized analyses of ACT efficacy data repository and the African Journals Online (AJOL) were used to confirm that no study was missed. Inclusion criteria were clinical trials conducted in Tanzania between 2001 and 2014 and involved at least one ACT for treatment of uncomplicated falciparum malaria. The studies should have reported the efficacy and/or safety of the tested drugs. The starting year (i.e., 2001) was purposely chosen because that was the year when WHO advocated use of ACT for treating uncomplicated falciparum malaria [5]. References were imported into the Endnote library and duplicates removed. An electronic matrix was developed in Microsoft Excel followed by full text review with predetermined criteria. The selected studies were each given an identification number, independently assessed for key information on efficacy and safety of ACT, which was extracted and summarized in tables and texts.

## Results

**Figure 1:**
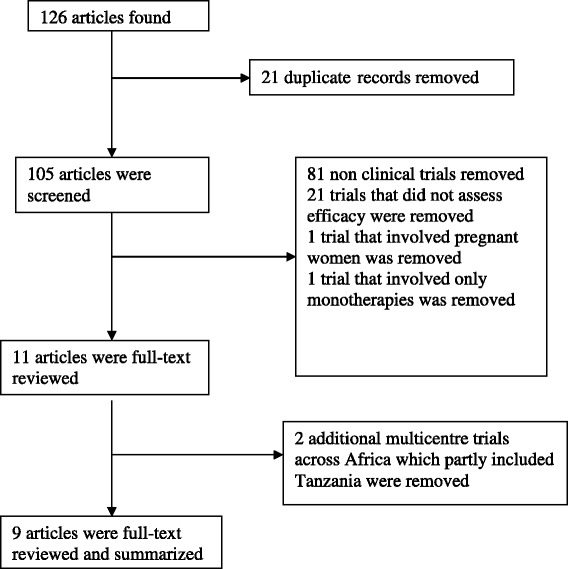
**Flow diagram for the literature search**

Literature search yielded 126 records, and 21 of these were duplicate records which were removed. The titles and abstracts of the remaining records (105) were screened based on the inclusion criteria and 11 articles qualified for a full-text review. From the review, two additional papers were from multicentre trials across Africa, which partly included Tanzania, and these were also removed. Only nine papers were left and fully reviewed as summarized in Figure 1.

### Studies conducted to test the efficacy of ACT in Tanzania

**Figure 2:**
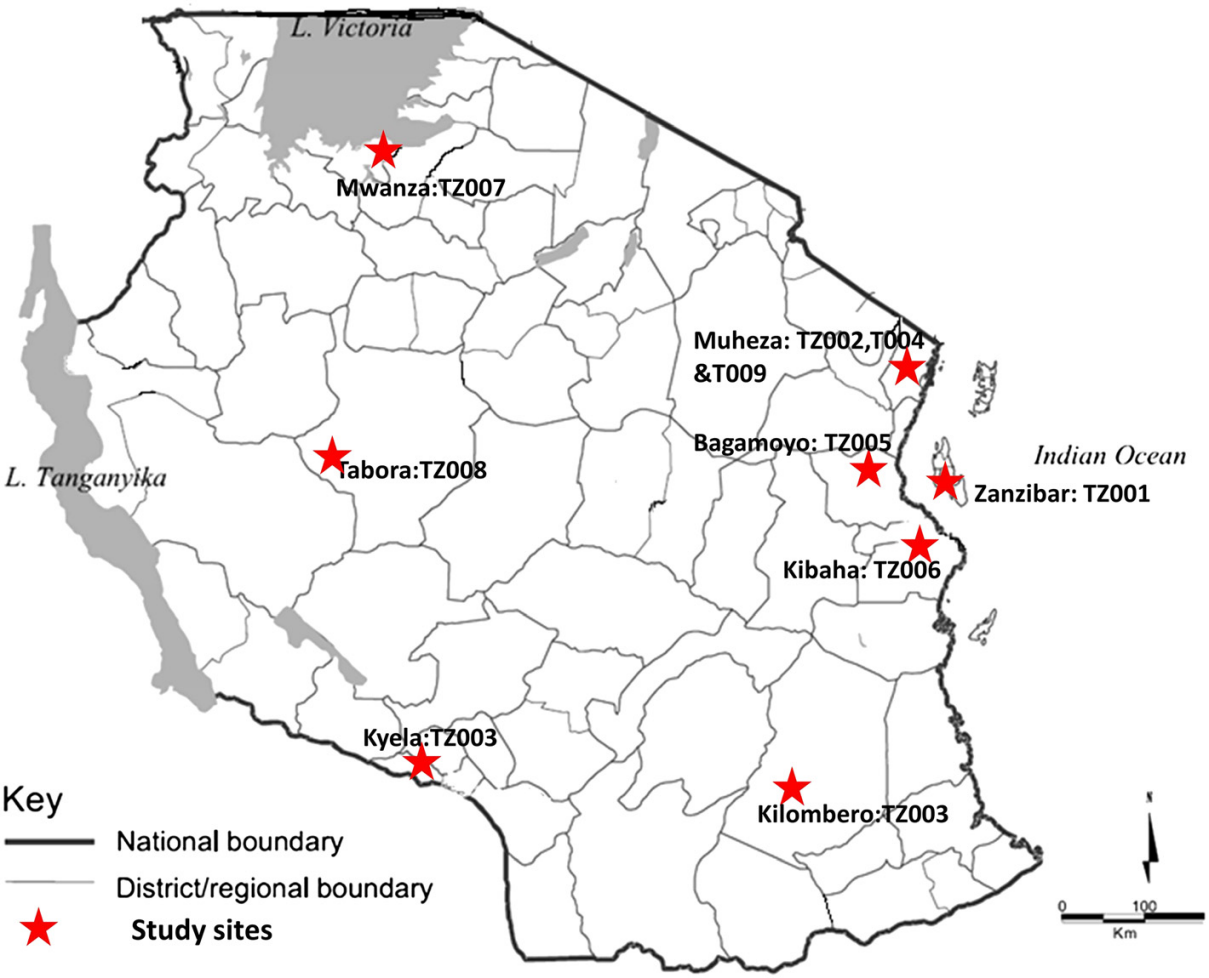
**Map of Tanzania showing the location at which the reviewed studies were conducted.**

Before and after the official adoption of ACT for treatment of uncomplicated malaria in Tanzania in 2007 [24], nine clinical trials were conducted in the country to assess the efficacy and/or safety of ACT for treatment of uncomplicated falciparum malaria (Table 1). All except three studies were conducted in the eastern part of Tanzania, with only one study from Zanzibar (Figure 2). Of the nine studies, three were conducted before the official adoption of ACT in Tanzania while the other six were undertaken thereafter (Table 1). All of the nine trials included AL testing, while five of these tested AL alone [34-38], three (37.5%) tested AL with AS + AQ [30,39,40], and one trial tested AL with azithromycin (AZ) [41]. Six of the trials that tested AL were randomized trials with more than one arm (Table 1).

**Table 1:** **Study design and baseline characteristics of efficacy trials that assessed AL and AS + AQ**

**Study ID**	**Authors**	**Year of study**	**Study duration in months**	**Study site**	**Randomization**	**Sample size**	**Days of follow-up**	**Parasitaemia inclusion criteria/**μ**l**	**Pf- GMPD**	**Supervised**
TZ001_AL	Martensson et al. [31]	2002-2003	4	Zanzibar	Yes, two arms	200	42	2,000-200,000	13,731	Yes
TZ001_ AS + AQ	Martensson et al. [31]	2002-2003	4	Zanzibar	Yes, two arms	208	42	2,000-200,000	19,731	Yes
TZ002_AL	Mutabingwa et al. [40]	2002-2004	26	Muheza	Yes, four arms	519	28	≥2,000	19,280	No
TZ002_ AS + AQ	Mutabingwa et al. [40]	2002-2004	26	Muheza	Yes, four arms	515	28	≥2,000	18,920	No
TZ003_AL	Kabanywanyi et al. [39]	2004	7	Kyela	Yes, four arms	99	28	2,000-200,000	43,115	Yes
TZ003_AS + AQ	Kabanywanyi et al. [39]	2004	7	Kilombero	Yes, four arms	76	28	2,000-200,000	49,348	Yes
TZ004_AL	Sykes et al. [41]	2008	7	Muheza	Yes, two arms	132	42	2,000-200,000	24,280**	Partial
TZ004_AZ + AS	Sykes et al. [41]	2008	7	Muheza	Yes, two arms	129	42	2,000-200,000	20,960**	Partial
TZ005_AL	Ngasala et al. [36]	2007-2008	12	Bagamoyo	Yes, two arms	180	56	2,000-200,000	41,885	Yes
TZ005*_AL	Ngasala et al. [36]*	2007-2008	12	Bagamoyo	Yes, two arms	179	56	2,000-200,000	38,272	No
TZ006_AL	Ngasala et al. [37]	2007	6	Kibaha	No, single arm	244	42	NR	19,054	No
TZ007_AL	Kamugisha et al. [35]	2010-2011	12	Mwanza	No, single arm	108	28	2,000-200,000	5,608	Yes
TZ008_AL	Joseph et al. [34]	2011	3	Tabora	No, single arm	20	28	2,000-200,000	39,400	Partial
TZ009_AL***	Shayo et al. [38]	2013	2	Muheza	No, single arm	88	28	250-200,000	18,603	Yes

Doses of AL were given twice a day for three days; Doses of AS + AQ were given once a day for three days;

TZ005_AL was the same study split into supervised and unsupervised*arm; *NR* = Not reported, Pf-GMPD = *P. falciparum* geometric mean parasite density of asexual parasites per microlitre of blood. **median parasite counts (asexual parasites per microlitre of blood) reported

***The study was conducted after malaria transmission had declined in the area and recruited children aged six months to 10 years and low cut-off of parasite density (250 asexual parasites/μl).

### Treatment outcome in studies that reported the efficacy of ACT in Tanzania

For the trials that tested AL, the reported PCR-corrected cure rates ranged from 91 to 100% (Table 2). The highest cure rate (100%) was reported by the studies conducted in Kyela in 2004, Tabora in 2011 and Muheza in 2013, while the lowest (91%) was reported in Muheza in 2008. PCR-corrected cure rates of AS + AQ ranged from 88.8 to 93.8% (Table 2). The PCR-corrected cure rate of AZ + AS was 68%. For the studies that tested AL when administered under supervision or unsupervised, the cure rates were comparable in the two groups. Although the study that tested the efficacy of AL when given unsupervised reported that more than half of the patients had recurrent infections within the 42-day follow-up period, the majority of these were due to re-infections and the cure rate was not significantly different when compared to the supervised arm. Studies that compared the efficacy of AL and AS + AQ showed a significantly lower risk of re-infection after treatment with AL than after treatment with AS + AQ [30,40].

**Table 2:** **Treatment outcome reported in efficacy trials in Tanzania**

**Study ID**	**Day-28 PCR-corrected % cure rate (95% CI)**
TZ001_AL	97.0 (NR)
TZ001_AS + AQ	91.0 (NR)
TZ002_ AL	97.2 (NR)
TZ002_ AS + AQ	88.8 (NR)
TZ003_ AL	100.0 (NR)
TZ003_ AS + AQ	93.8 (NR)
TZ004_ AL	91.0 (NR)
TZ004_ AZ + AS	68.0 (NR)
TZ005_ AL	98.8 (95.5-99.7)
TZ005*_ AL	98.2 (94.5-99.4)
TZ006_ AL	95.1 (91.4-97.7)
TZ007_ AL	96.0 (NR)
TZ008_ AL	100.0 (NR)
TZ009_ AL	100.0 (NR)

TZ005_AL was the same study split into supervised and unsupervised*arm.

95% *CI* = 95% Confidence interval;

*NR* = Not reported.

### Fever, parasite and gametocyte clearance in studies that reported efficacy of ACT in Tanzania

Among the five supervised trials that reported fever clearance, more than 80 and 79% of the patients cleared fever by day 1 post-treatment with AL and AS + AQ, respectively. None of the patients had fever on day 3 (Table 3). Among the five studies that reported parasite clearance, two studies showed day 3 parasitaemic cases of 1.1 and 1.4% (Table 3).

**Table 3:** **Fever and parasite clearance in efficacy trials in Tanzania**

**Study ID**	**Fever clearance by D1 (%)**	**Day 1 Parasitaemic (%)**	**Day 2 Parasitaemic (%)**	**Day 3 Parasitaemic (%)**
TZ001_AS + AQ	79.0	66.0	10.0	0
TZ001_AL	67.0	83.0	10.0	0
TZ005_ AL	64.3	71.3	6.7	1.1
TZ007_ AL	95.0	32.0	11.7	0
TZ008_ AL	95.0	NR	0	0
TZ009_ AL	73.9	77.3	19.5	1.4

D1refers to day 1;

*NR* = Not reported.

In the present review, four papers reported gametocyte carriage [30,34,37,40] and the proportion of patients with gametocytes was significantly reduced from that recorded during enrolment compared to what was reported after treatment with AL or AS + AQ. Joseph et al. [34] showed an unusual increase in gametocytes in one patient treated with AL, from four on day-0 to 68 sexual parasites per 500 leucocytes on day-2.

### Day-7 plasma lumefantrine levels

Three studies measured the median day-7 lumefantrine levels [34,36,37] and one of these showed that the median plasma lumefantrine concentration was significantly higher in the supervised than in the unsupervised group (P <0.001) [35]. Furthermore, the median day-7 plasma lumefantrine concentration was significantly lower in patients with recrudescence compared to those with re-infections or no parasite re-appearance [38]. It was further shown that lumefantrine concentration at day 7 tended to decrease with a unit increase in weight (kg), although the decrement was not statistically significant [34].

### Safety profile of ACT reported in clinical trials conducted in Tanzania

The safety of ACT was assessed by recording the nature and incidence of solicited and unsolicited adverse events and serious adverse events. An adverse event was defined as any undesirable medical occurrence (symptoms, signs or laboratory findings) in a patient during the study regardless of whether it was related to the treatment. Adverse events were judged according to their causal association with ACT (unlikely, possible and probable) and severity (mild, moderate or severe) [33]. Cough was the most common adverse event among children treated with AL, while severe malaria was the most reported serious adverse event requiring hospitalization among children treated with AL or AS + AQ (Table 4). Other adverse events (as summarized in Table 4) were mild and not directly attributed to the treatment.

**Table 4:** **Adverse events reported in efficacy trials in Tanzania**

**Study ID**	**Adverse events reported**	**Comments**
TZ001_AS + AQ	Severe malaria (3.4%)	Not attributed to the treatment
TZ001_AL	Severe malaria (1.0%)	Not attributed to the treatment
TZ002_ AL	Death (0.2%)	Not attributed to the treatment
TZ004_ AL	Gastrointestinal complaints (5.3%), vomiting (1.5%), dermatological (including itching) (3.0%), respiratory (including respiratory infection) (15.9%), dizziness (0.8%), convulsions (2.3%)	Gastrointestinal complaints were likely to be attributed to the drugs
TZ004_ AZ + AS	Gastrointestinal complaints (2%), vomiting (7.6%), dermatological (including itching) (5.4%), respiratory tract respiratory infections (11.6%), dizziness (1.6%), convulsions (0.8%)	Gastrointestinal complaints were likely to be attributed to the drugs
TZ005_ AL	Severe malaria (4%), vomiting (1%), cough (10%), abdominal pain (1%), diarrhoea (3%), weakness (1%), upper respiratory tract infections (22%), skin infections (9%), urinary tract infection (5%), otitis media (4%), tonsillitis (2%), conjunctivitis (4%), worm infestation (1%), periodontitis (1%), asthma (0.3%)	Severe malaria was recorded as severe adverse event. The rest were mild or moderate in severity
TZ006_ AL	Severe malaria (1%), fever (34%), cough (34%), diarrhoea (12%)	Severe malaria was recorded as severe adverse event. The rest were mild or moderate in severity. None was considered related to AL treatment
TZ009_ AL	Cough (49.4%), fever (20.2%), abdominal pain (10.1%), diarrhoea (1.3%), Headache (1.3%), skin rashes (1.3%)	No serious adverse events. All the AEs were not related to the treatment

## Discussion

Following the recent reports of emergence of *P. falciparum* artemisinin-resistant field isolates in Southeast Asia [1,2,19] and the threat of such parasites spreading to other malaria-endemic countries, country-specific evidence based on reliable data are urgently required to monitor the efficacy of the drugs and support timely review and implementation of malaria treatment guidelines. Surveillance of anti-malarial efficacy is crucial to enable early detection of emergence of drug resistance when it happens before it spreads in most of the populations, as happened for CQ and SP [33]. Information generated from such surveillance provides evidence to relevant national and international authorities for policy formulation and review. This review was undertaken to assess the implementation of efficacy testing for monitoring of therapeutic efficacy of ACT for treatment of uncomplicated falciparum malaria in Tanzania before and after policy changes (in 2006).

Apparently, due to limited resources, especially funding, NMCPs in most endemic countries have not been able to implement regular anti-malarial drug efficacy monitoring at sentinel sites and there has been a strong call for regional networks to facilitate the implementation [42]. The former East African Network for monitoring anti-malarial treatment (EANMAT) forged a partnership between the ministries of health and the research community in East African countries and facilitated monitoring of anti-malarial drug resistance in the region [28]. However, EANMAT collapsed as a result of many factors, including dependence on short-term donor funding.

The findings of this review showed that nine clinical trials have been conducted to monitor the efficacy of ACT before and after Tanzania adopted ACT for treatment of uncomplicated malaria. Of these, only one study [39] was conducted within the in vivo efficacy testing framework of Tanzania NMCP/EANMAT with financial support from NMCP and EANMAT. This might be partly due to lack of funding, or due to complacency attributed to perceived high therapeutic efficacy of ACT. However, in Tanzania, NMCP-supported TETs have resumed since 2011, although the findings have not yet been published.

The present review has shown that the efficacy of AL, which is the first-line anti-malarial drug for treatment of uncomplicated falciparum malaria in Tanzania, was high even after unsupervised treatment. The PCR-corrected cure rate on day 28 was >91% and this is in line with findings from other studies in eastern Africa [43,44]. The high cure rate (100%) reported in Muheza in 2004 [39] prior to the official adoption of AL was similar to the cure rate reported in other African countries [45,46]. The efficacy of AS + AQ, which is currently the first-line anti-malarial drug for treatment of uncomplicated falciparum malaria in Zanzibar (an island part of the United Republic of Tanzania) [30], was also high although comparably lower than that of AL (PCR-corrected cure rate was 88.8 - 93.8% for AS + AQ compared to 91 -100% for AL). The lower cure rates of AS + AQ compared to AL could be attributed to the fact that AQ had been extensively used in the country and was also adopted as a second-line anti-malarial drug together with SP (which was the first-line) in 2001 [25]. Since AQ resistance had been reported in Tanzania [27,47], addition of an artemisinin to AQ was unlikely to make a combination with high therapeutic efficacy. Similar failure rates have been reported in other SSA countries that used AQ extensively prior to introduction of AS + AQ [48].

The cure rates of AQ + AS in the present review compares well with those reported elsewhere in East Africa whereby day-28 adequate clinical and parasitological response (ACPR) in children treated with AS + AQ was 90.2% in Kenya [49], 90.3% in Rwanda [50] and 91.7% in Uganda [51]. These rates were comparatively lower than those of AL. However, AS + AQ was selected and is still being used as the first-line anti-malarial in some countries when other factors apart from efficacy were considered. In Burundi, prior to policy changes, day-14 ACPR in children treated with AS + AQ was reported to be 95.3% compared to 99.3% for AL. However, considering other factors such as acceptability by users, adherence and cost, AS + AQ was chosen as the first-line anti-malarial for treatment of uncomplicated falciparum malaria in Burundi [10,12]. Similarly, Zanzibar adopted AS + AQ as the first-line anti-malarial despite lower day-28 ACPR compared to AL (97% versus 91% for AS + AQ) [30].

There is a concern about the limited post-treatment prophylactic effects of both AL and AS + AQ in high transmission areas. In fact in one trial, more than half of the recruited patients had recurrent infections within the 42-day follow-up period after treatment with AL. However, the majority of recurrent infections were due to re-infections which suggests that the partner drug cannot give prolonged protection despite high therapeutic efficacy [36]. Similar high re-infections rates have been reported in other high transmission areas in Africa after AL treatment [43,52]. Studies that compared the efficacy of AL and AS + AQ showed a significantly lower risk of re-infection after treatment with AL compared to AS + AQ [30,40] suggesting that AL confers a longer prophylactic effect than AS + AQ. The difference in prophylactic effect of the two drugs could be attributed to the longer half-life of lumefantrine compared to AQ. Thus, the concentration of the active amodiaquine metabolite might be lower or completely absent when a re-infection occurs compared to lumefantrine concentration. This observation has also been reported elsewhere in Africa where re-infection rates were higher after AS + AQ treatment than after AL [48,53,54]. However, a recent study has reported high level of resistance to lumenfatrine in the Democratic Republic of Congo [55] that threatens the therapeutic usefulness of AL and further monitoring is urgently needed in all malaria-endemic countries where AL is the first-line anti-malarial drug.

In most of the studies, a great majority of the recurrent infections were due to re-infections, when assessed with a step-wise PCR genotyping protocol, which signifies that the drugs are still efficacious and the high rates of re-infections could only be attributed to high malaria transmission. In terms of clinical practice, the high re-infection rates are of great concern among clinicians. Clinicians should be clearly guided on what to expect and how to handle such cases with recurrent infections within a period of three to eight weeks post-treatment. The observed high re-infection rates after ACT treatment underscores the importance of integrating treatment with vector control interventions, including use of long-lasting insecticide-treated nets so as to effectively block malaria transmission and prevent recurrent infections [56].

The study which tested AZ + AS showed that the drug had low efficacy (28 days ACPR = 68%) and could not be considered a potential anti-malarial drug in Tanzania and other malaria-endemic countries [41]. It is plausible that since AZ is a common antibiotic in the treatment of trachoma, the local parasites might have been exposed to the drug leading to development of resistance [57]. This could have possibly compromised the efficacy of AZ + AS combination. An alternative explanation for the observed lower efficacy of AZ + AS compared to adults in Asia is that the effective dose of AZ absorbed in often-malnourished African children might not be sufficient to achieve adequate cure rates. Malnourishment is known to reduce drug absorption [8] and cure rates among patients treated with different drugs. Furthermore, a recent review of AZ across continents for treating uncomplicated malaria revealed that AZ has low efficacy as a monotherapy for treatment of uncomplicated malaria and when used in combinations with other anti-malarials, it may need to be used at high doses which may affect tolerability to the drug [58].

Measurement and reporting of parasite clearance on day 3 after treatment with ACT is particularly important, as this is one of the first signals of emergence of parasite tolerance/resistance to artemisinin [23]. In the present review, two studies reported day-3 parasitaemic cases of 1.1 and 1.4% after treatment with AL [36,38] and the day 3 parasite positivity rates were lower than what has been previously reported [21]. However, the parasite positivity rate reported on day 2 in one of the studies conducted in Muheza district with moderate malaria transmission was higher than the rates reported in previous studies [21]. Thus, more studies will be required to confirm these findings and their role in possible emergence of artemisinin resistance. Although the proportion of patients with detectable parasitaemia on day 3 serves as a simple measure of parasite clearance time at the population level [21], it is often influenced by the baseline parasite density and the timing of parasite sampling, which can vary within and across studies. On the contrary, parasite clearance half-life doesn’t depend on baseline parasite density and is thus considered a more reliable indicator of changes in parasite susceptibility to artemisinin. Measurement of parasite clearance at six-, eight- or 12-hourly intervals for the first 72 hours, as it is currently recommended [59,60], provides a population level profile and useful data of parasite sensitivity to artemisinin. More accurate estimates of parasite clearance through frequent parasite counts are recommended [59,60]. However, the studies reviewed in this article were based on 24-hour sampling, which is not the recommended method for assessing parasite clearance and detection of tolerance/resistance to artemisinins.

Artemisinins are known to be highly potent anti-malarial drugs that are active against immature gametocytes and are useful in the reduction of malaria transmission and elimination/eradication agenda [61]. In clinical trials reviewed in this paper, it was shown that in fact AL and AS + AQ have potentials to reduce gametocyte carriage [30,34,37,40]. However, the unusual increase in gametocytes from four on day-0 to 68 sexual parasites per 500 leucocytes on day-2 post AL treatment, as reported in one of the trials [34], needs to be further evaluated in the light of changes in the parasite sensitivity to ACT. Gametocyte clearance by ACT has also been documented by other studies in East Africa [62] and elsewhere [63,64] where significant reduction of gametocytes by day 14 after treatment with AL or AS + AQ was observed, indicating the potential advantages of ACT over non-artemisinin-based anti-malarials.

It is well established that the efficacy of AL combination is strongly influenced by variations in the pharmacokinetics of lumefantrine among individuals [8]. The maximum therapeutic cure rate is achieved when the plasma drug concentration is adequately available for at least six days [65]. Measurements of day-7 plasma lumefantrine levels are particularly important in unsupervised trials as a measure of adherence to treatment, rather than the use of questionnaires [66]. Day-7 lumefantrine concentrations were significantly lower in unsupervised patients suggesting lower adherence to the drug dosage or fat intake advice. However these differences did not affect the cure rates and high therapeutic efficacy was achieved even in the unsupervised group, indicating that the parasites are highly susceptible to lumefantrine. The observation by other studies in East Africa which showed the median lumefantrine levels were significantly lower in unsupervised patients, but without any effects on the cure rates [67,68], lends support to the findings of this review. It is clear that a high day-28 AL cure rate can be achieved despite low plasma lumefantrine levels, even among unsupervised patients. However care should be taken to avoid exposure of parasites to sub-therapeutic levels of the drugs and creating favourable conditions for emergence of lumefantrine resistance [69]. Monitoring of lumefantrine tolerance/resistance should also be implemented in order to safeguard usefulness of AL.

The present review showed no unexpected adverse events and overall, AL, AS + AQ and AZ + A were well tolerated. Admittedly, the few studies that reported safety profile in the present review (e.g., only one study reported safety data on AS + AQ while the rest reported AL safety) would not enable a firm comparison of safety of different anti-malarials. However, other studies in Africa have shown that certain mild or moderate adverse events, such as vomiting and anaemia, were more frequent in patients treated with AS + AQ than those treated with AL [70,71]. This review has shown that respiratory infection, including cough, was the most frequent adverse event in children treated with AL. This is in line with previous findings which showed that respiratory infections were common in African children with malaria [72,73]. The findings from a recent review on the safety of AL with other ACT in children [73] showed that adverse events were attributed to symptoms or progression of malaria and not directly to the drugs, and this lends support to the findings of this review. Thus, AL and AS + AQ are safe when used for treatment of uncomplicated malaria.

### Limitation of the review

This review was meant to assess the implementation of the WHO recommendations of undertaking regular monitoring of antimalarial efficacy studies and also provide Tanzania-specific current efficacy and safety profile of ACT in the treatment of uncomplicated falciparum malaria. The review was limited to peer-reviewed publications, thus unpublished data were not included. However the review highlighted the levels of implementation of TETs in Tanzania and provides an overall country-specific performance of ACT after their wide-scale deployment as first-line anti-malarials for treating uncomplicated *P. falciparum* malaria in the country.

### Future studies

Following the emergence of artemisinin resistance in Southeast Asia, manifested as delayed clearance of *P. falciparum* after treatment with artemisinins, the demand for tracking parasite sensitivity to artemisinin and its derivatives has become more important. More accurate estimates of parasite clearance measurements through frequent parasite counts (at least twice daily) to assess delayed parasite clearance should be adopted in future therapeutic efficacy testing studies [59,60]. However these changes have significant cost and logistic implications that must be addressed.

Given the recent documentation of K13-propeller polymorphism as a molecular marker for monitoring resistance of artemisinin and its derivatives [22] and despite absence of Asian mutant genotypes in SSA [74], future efficacy studies should incorporate assessment of this marker as a tool to track parasite tolerance or any changes in parasite sensitivity to ACT [75]. Furthermore, the recent report of high resistance to lumefantrine should also be assessed in both in vivo and in vitro studies. More importantly, optimization and testing of other methods for resistance surveillance such as ex-vivo and ring stage assay should be considered for future studies in SSA.

## Conclusion

The present review has shown that few studies have been conducted in Tanzania to monitor the efficacy and safety of ACT and majority of these were not done under the NMCP framework. However, the findings revealed that the efficacy AL and AS + AQ was reasonably high and the drugs were safe when used for treatment of uncomplicated *P. falciparum* infections in Tanzania. These findings support continued use of AL and AS + AQ for the treatment of uncomplicated malaria in Tanzania mainland and Zanzibar, respectively. Although currently there is no evidence of artemisinin resistance in Africa, regular monitoring and surveillance, as recommended by the WHO-supported GPARC must be implemented so that the emergence of artemisinin resistance in African can be timely detected, reported and contained. More surveillance and monitoring of anti-malarial efficacy and safety should be performed to detect future changes in parasite sensitivity to ACT.

## Abbreviations

AS + AZ: Artesunate azithromycin
ACT: Artemisinin combination therapy
AE: Adverse event
AL: Artemether-lumefantrine
AS + AQ: Artesunate-amodiaquine
SAE: Serious adverse event
CQ: Chloroquine
SP: Sulphadoxine/pyrimethamine
DHA + PQ: Dihydroartemisinin-piperaquine
AS + SP: Artesunate-sulphfadoxine/pyrimethamine
NMCP: National malaria control programme
TETs: Therapeutic efficacy trials
SSA: Sub-Saharan Africa
GPARC: Global plan for artemisinin resistance containment
EANMAT: East African network for monitoring anti-malarial treatment
WHO: World Health Organization
